# Serological investigation of zoo animals: SARS-CoV-2 antibodies detected in a European moose and an Asian golden cat

**DOI:** 10.1186/s42522-026-00227-2

**Published:** 2026-07-01

**Authors:** Kerstin Wernike, Dominik Fischer, Lisa Grund, Martin Beer

**Affiliations:** 1 Friedrich-Loeffler-Institut, Südufer 10, 17493 Greifswald - Insel Riems, Germany; 2 Der Grüne Zoo Wuppertal, Hubertusallee 30, 42117 Wuppertal, Germany

**Keywords:** COVID-19, Coronavirus, Serology, Zoo animals, Reservoir

## Abstract

As the human COVID-19 pandemic progressed, the causative agent severe acute respiratory syndrome coronavirus 2 (SARS-CoV-2) was detected in an increasing number of animal species, raising concerns about the establishment of new animal reservoirs. Here, we investigated the presence of SARS-CoV-2 antibodies in serum samples of 123 mammals housed in various zoological institutions in Germany with direct or indirect contact to humans (visitors and zoo staff), collected between December 2019 and January 2025. The animals included diverse representatives from the orders Artiodactyla (*n* = 59), Carnivora (*n* = 54), as well as Proboscidea (*n* = 7), two perissodactyls, and a rodent. All serum samples were tested using a species-independent commercial surrogate virus neutralization test (sVNT) and a multispecies receptor-binding domain (RBD)-based ELISA. With two exceptions, all samples tested consistently negative in both assays. However, antibodies against SARS-CoV-2 were detected in a European moose (*Alces alces*) and an Asian golden cat (*Catopuma temminckii*), sampled in 2020 and 2021, respectively, expanding the known host range of SARS-CoV-2. Hence, pathogen monitoring and continued health surveillance in both captive and wild animal populations are essential for understanding the actual host range, potential reservoir formation, and the broader implications for animal and public health.

## Main text

The severe acute respiratory syndrome coronavirus 2 (SARS-CoV-2), which was first identified in late 2019 in Wuhan, China [1], spread very rapidly worldwide driven by human-to-human transmission through respiratory droplets and aerosol, leading to the COVID-19 pandemic and millions of deaths worldwide [2, 3]. While initially thought to be a purely human health concern, the virus was found in an increasing number of animal species as the pandemic progressed [4, 5], raising concerns about long-term viral persistence and the establishment of animal reservoirs also due to anthropozoonotic transmission. In addition to domestic pets such as cats and dogs and farm animals with close contact to humans, SARS-CoV-2 has been detected in various zoo animals, particularly in large cats such as lions and tigers, non-human primates, and some cloven-hoofed animals [5–19], typically following contact with infected humans. In addition, the virus or specific antibodies have been found in several wild mammals, among them artiodactyls, with growing concern about large-scale virus spread among free-ranging deer populations, in particular white-tailed deer (*Odocoileus virginianus*) [20–24]. While white-tailed deer have been the primary focus of research, experimental infection studies suggest that other large ruminants, such as North American elk (*Cervus elaphus canadensis*) or mule deer (*Odocoileus hemionus*), may also be susceptible [25, 26]. However, the full host range is presumably not yet known. Since understanding how SARS-CoV-2 behaves in non-human hosts is crucial for managing risks to animal health, biodiversity, and public health, we have examined animals, with a special focus on artiodactyls and carnivores, kept in human care in zoological institutions (zoos, wildlife parks, theme parks) in Germany. The institutions were located in the German federal states of North Rhine-Westphalia (Zoo Wuppertal, Wildfreigehege Hellenthal), Lower Saxony (Wildpark Schwarze Berge) and Baden-Württemberg (Wildparadies Erlebnispark Tripsdrill).

**Fig. 1:**
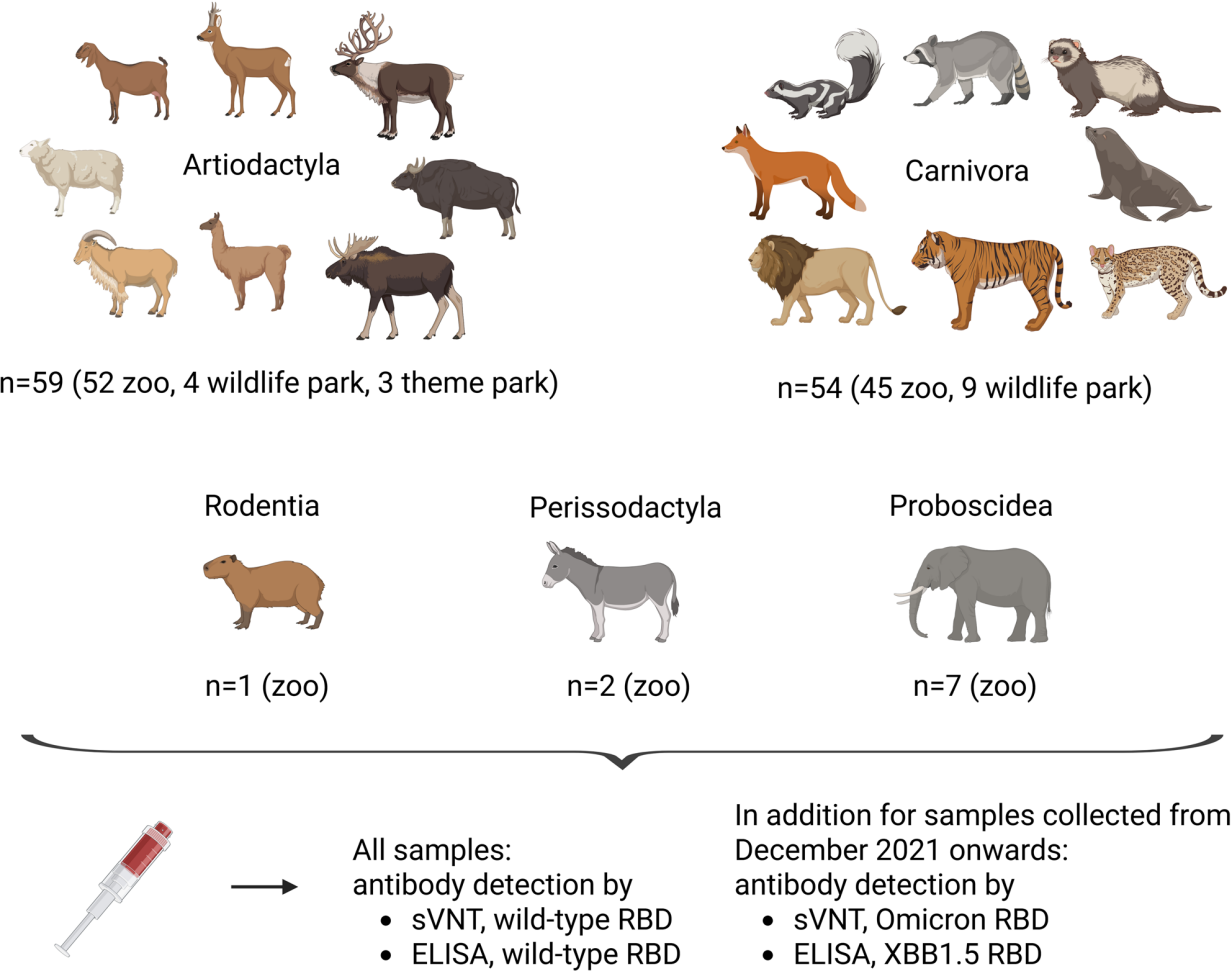
Experimental design. Blood samples of 123 animals from the orders Artiodactyla, Carnivora, Rodentia, Perissodactyla and Proboscidea kept in zoological institutions in Germany were collected (upper panel) and analyzed for SARS-CoV-2 antibodies (lower panel). All samples were tested by a commercial surrogate virus neutralization test (sVNT) and a multispecies receptor-binding domain (RBD)-based ELISA. Samples collected from December 2021 onwards were also tested in a parallel approach against an Omicron ortholog of the RBD-protein in both tests

In total, blood samples from 123 individuals taken between December 2019 and January 2025 were available from continuous health monitoring or other studies (Supplementary table in the Zenodo repository). Fifty-nine of the animals belong to the order Artiodactyla and 54 to the order Carnivora. In addition, seven African elephants (order Proboscidea), two perissodactyls and one rodent were included (Fig. 1). All sera were tested by a commercial, species-independent surrogate virus neutralization test (sVNT) (cPass™ SARS-CoV-2 Neutralization Antibody Detection Kit, GenScript, the Netherlands) and by a multispecies RBD-based ELISA [27]. The sVNT was performed according to the manufacturer’s instruction using a serum dilution of 1/10 and a cutoff of ≥ 30% inhibition for positivity and < 30% for negativity. The ELISA was carried out as described previously using a serum dilution of 1/100 and a cutoff of ≥ 0.3 of the optical density (OD) measured at 450 nm for positivity [27]. The original composition of both tests allows for the detection of antibodies against the wild-type virus and the variants of concern up to Delta. The samples collected from December 2021 onwards, i.e. after the emergence and global spread of Omicron variants, were also tested in a parallel approach against an Omicron ortholog of the receptor-binding domain (RBD) protein in both tests. For the sVNT, an Omicron-specific horseradish peroxidase-(HRP-) conjugated RBD is provided by the test manufacturer and the suitability to detect Omicron-specific antibodies in animals has been demonstrated [28]. In the multispecies ELISA, the Omicron XBB1.5 variant protein was used [29].

**Fig. 2:**
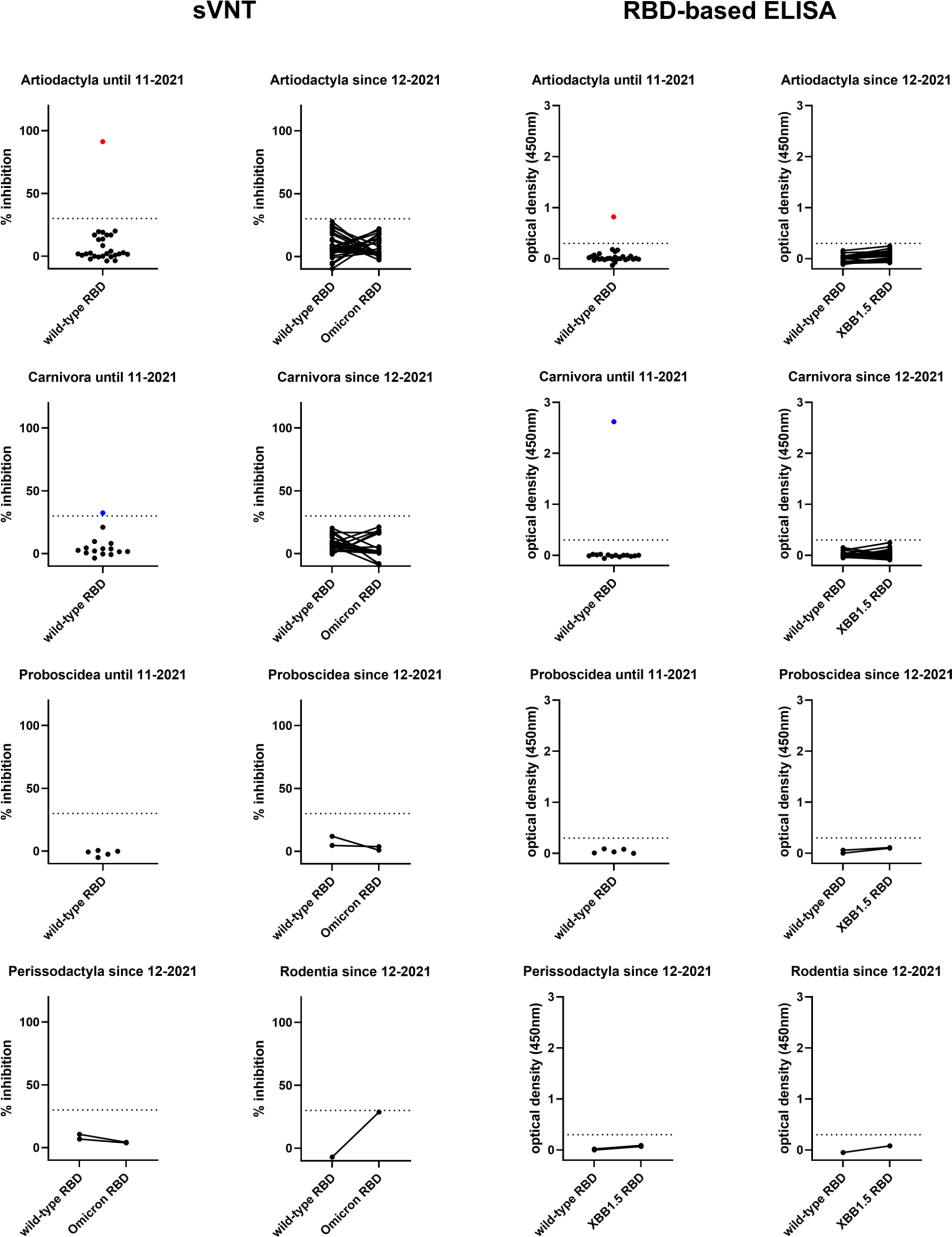
Results of the SARS-CoV-2 serological tests. The results of the surrogate virus neutralization test (sVNT) are displayed in the two columns on the left, while those of the multispecies receptor-binding domain (RBD)-based ELISA are shown in the two columns on the right. The results are presented separately for the samples taken up to November 2021 and the samples taken since December 2021, as the latter were additionally tested against the Omicron ortholog of the RBD-protein in both tests. The cutoff values of the respective tests are shown by dotted lines. The values of the blood sample from the European moose that tested positive in both, sVNT and RBD-ELISA, are highlighted in red and the values of the Asian golden cat are highlighted in blue

With two exceptions, all samples reacted negative in both tests, and when the Omicron ortholog of the RBD was additionally applied in the sVNT and ELISA, also against this protein (Fig. 2). The exceptions were a European moose (*Alces alces*, order Artiodactyla) kept in a wildlife park and a female Asian golden cat (*Catopuma temminckii*, order Carnivora) kept in a zoo. The European moose was sampled in 2020 and tested positive in both wild-type RBD-based assays (sVNT 91.21% and ELISA OD 0.82). In the Asian golden cat, which was sampled in 2021, antibodies against SARS-CoV-2 were likewise detected in both tests using the wild-type RBD (sVNT 32.40% and ELISA OD 2.62) (Fig. 2).

The detection of SARS-CoV-2 antibodies in the European moose and the Asian golden cat from zoological institutions in Germany provides valuable insights into the broader host range of the virus and highlights the potential for spillover events into non-human animal populations, even under managed care. These findings contribute to the growing body of evidence that SARS-CoV-2 is capable of infecting a wide range of mammalian species, both domesticated and wild [5, 30], and raise important questions about the virus’s long-term ecological and epidemiological implications. Interestingly, the samples in which SARS-CoV-2 antibodies were detected in this study, have been taken in 2020 and 2021 and therefore predate the emergence and global dominance of Omicron variants. After 2021, reports of SARS-CoV-2 infections in animals generally have markedly decreased, potentially related to varying susceptibilities to the circulating virus variants. For the domestic carnivores cat and dog, a field study in Germany comparing seroprevalence before and during the transition from Delta to Omicron circulation found a marked decrease in the proportion of seropositive animals after late 2021, suggesting that dogs and cats have considerably lower susceptibility to the Omicron VOC [28]. However, it is not yet known whether this also applies to nondomestic zoo or wild carnivores.

Particular worrying with regard to the possible formation of animal reservoirs are the situations in free-ranging white-tailed deer and farmed mink (order Carnivora). In white-tailed deer, widespread infections have been documented in several regions, with evidence of sustained deer-to-deer transmission and the presence of viral variants [20–24, 31, 32], suggesting that deer populations may serve as a long-term reservoir for the virus, potentially allowing it to evolve independently from human strains and possibly spill back into humans. In farmed mink, outbreaks have shown that the virus can spread rapidly within densely housed populations, leading to increased daily mortality rates and, critically, documented cases of mink-to-human transmission [33–37] and the spread of a mink-associated strain in the human community [38]. In addition to the aforementioned two species, a multitude of others have been demonstrated to be susceptible, encompassing diverse artiodactyl and carnivorous species among others. Here, we showed previous SARS-CoV-2 exposure by serological methods in two non-domestic species not commonly associated with human interaction or COVID-19 surveillance, thereby expanding the known susceptibility of species within the orders Artiodactyla and Carnivora. The negative results across the other 121 individuals from diverse mammalian taxa are reassuring in the sense that widespread, undetected SARS-CoV-2 circulation in captive animals in the investigated German facilities appears unlikely. However, the absence of evidence is not evidence of absence, as the sample sizes for certain taxa, particularly perissodactyls and rodents, were too small to draw any conclusions. Nevertheless, the detection of antibodies in a European moose supports the idea that not only white-tailed deer, but also Old World cervids, may be at risk, especially as some studies demonstrated antibodies also in fallow deer (*Dama dama*) and red deer (*Cervus elaphus*) [39, 40], though others have found no evidence for SARS-CoV-2 infection of fallow and red deer populations [41, 42]. Taking all available studies together, there is currently no evidence to suggest the establishment of additional ruminant wildlife reservoirs, with the notable exception of white-tailed deer in North America. However, further surveillance in free-ranging European wildlife or experimental infection studies are required to study the actual susceptibility and possibly the viral spread in the field.

Given the character of our investigation, it is not possible to make any statements regarding the potential clinical manifestations of infection in the animals under study. The detection of antibodies, rather than active virus, suggests that the European moose and the Asian golden cat were infected weeks or months prior to sampling, but the exact timepoint is unknown and thus no conclusions can be drawn about their health status at the time of infection. The source of the infection of the animals remains also undetermined. Nevertheless, considering the high infection rates in humans in Germany in the relevant period [43] and acknowledging the prior studies conducted in various zoological institutions that indicated transmission through close contact with infected humans, such as zookeepers [10, 12, 13, 15], it can be hypothesized that infected humans may have served as the source of infection for the two animals in our case as well. The fact that only two animals were found to be seropositive suggests that there were only isolated spillover infections and that no chains of infection formed in the zoological facilities examined. Nevertheless, in general, human-animal contact networks in zoological settings and other contexts involving animal-human interactions, such as agriculture, should be assessed in order to identify potential pathways of virus introduction into the animal populations. Therefore, continued monitoring, particularly in susceptible taxa like artiodactyls and carnivores, is critical not only for animal health and conservation but also for anticipating and preventing future zoonotic events.

## Data Availability

The datasets generated and analyzed during the current study are available in the Zenodo repository, DOI: 10.5281/zenodo.209726.

## Abbreviations

HRP: Horseradish peroxidase
OD: Optical density
RBD: Receptor-binding domain
SARS-CoV-2: severe acute respiratory syndrome coronavirus 2
sVNT: Surrogate virus neutralization test
